# Central projections of antennular chemosensory and mechanosensory afferents in the brain of the terrestrial hermit crab (*Coenobita clypeatus*; Coenobitidae, Anomura)

**DOI:** 10.3389/fnana.2015.00094

**Published:** 2015-07-15

**Authors:** Oksana Tuchina, Stefan Koczan, Steffen Harzsch, Jürgen Rybak, Gabriella Wolff, Nicholas J. Strausfeld, Bill S. Hansson

**Affiliations:** ^1^Department of Evolutionary Neuroethology, Beutenberg Campus, Max Planck Institute for Chemical EcologyJena, Germany; ^2^Laboratory for Genomic and Proteomic Research, Institute of Chemistry and Biology, Immanuel Kant Baltic Federal UniversityKaliningrad, Russia; ^3^Cytology and Evolutionary Biology, Zoological Institute and Museum, Ernst Moritz Arndt University GreifswaldGreifswald, Germany; ^4^Department of Neuroscience, The University of ArizonaTucson, AZ, USA

**Keywords:** chemical ecology, terrestrialization, olfaction, hermit crabs, retrograde tracing

## Abstract

The Coenobitidae (Decapoda, Anomura, Paguroidea) is a taxon of hermit crabs that includes two genera with a fully terrestrial life style as adults. Previous studies have shown that Coenobitidae have evolved a sense of spatial odor localization that is behaviorally highly relevant. Here, we examined the central olfactory pathway of these animals by analyzing central projections of the antennular nerve of *Coenobita clypeatus*, combining backfilling of the nerve with dextran-coupled dye, Golgi impregnations and three-dimensional reconstruction of the primary olfactory center, the antennular lobe. The principal pattern of putative olfactory sensory afferents in *C. clypeatus* is in many aspects similar to what have been established for aquatic decapod crustaceans, such as the spiny lobster *Panulirus argus*. However, there are also obvious differences that may, or may not represent adaptations related to a terrestrial lifestyle. In *C. clypeatus*, the antennular lobe dominates the deutocerebrum, having more than one thousand allantoid-shaped subunits. We observed two distinct patterns of sensory neuron innervation: putative olfactory afferents from the aesthetascs either supply the cap/subcap region of the subunits or they extend through its full depth. Our data also demonstrate that any one sensory axon can supply input to several subunits. Putative chemosensory (non-aesthetasc) and mechanosensory axons represent a different pathway and innervate the lateral and median antennular neuropils. Hence, we suggest that the chemosensory input in *C. clypeatus* might be represented via a dual pathway: aesthetascs target the antennular lobe, and bimodal sensilla target the lateral antennular neuropil and median antennular neuropil. The present data is compared to related findings in other decapod crustaceans.

## Introduction

Besides Isopoda (Harzsch et al., 2011; Kenning and Harzsch, 2013), certain decapod crustaceans have succeeded in meeting the physiological challenges of a terrestrial life style (review Hansson et al., 2011) including the need for olfactory sensory systems to function in air rather than in water. Prominent amongst land crustaceans are members of the Coenobitidae (Decapoda, Anomura, Paguroidea), a taxon of hermit crabs that includes two genera with fully terrestrial life styles as adults, *Coenobita* and *Birgus* (Greenaway, 1999, 2003; McLaughlin et al., 2007; Drew et al., 2010). Field experiments have provided evidence that Coenobitidae have evolved a sense of distance olfaction that plays an essential role in guiding the animal's responses to environmental stimuli (Rittschof and Sutherland, 1986; Vannini and Ferretti, 1997; Stensmyr et al., 2005). Using electroantennogram recordings (EAG) it was possible to establish the response spectrum of olfactory sensory neurons in *Coenobita clypeatus* (Krång et al., 2012) and *Birgus latro* (Stensmyr et al., 2005) to various chemical stimuli such as number of simple organic acids, amines, aldehydes. Furthermore, analysis of the antennular transcriptome of *C. clypeatus* combined with *in situ* hybridization experiments revealed the absence of ligand-gated odorant receptors but the presence of ionotropic receptors (IRs) that are expressed in olfactory sensory neurons and most likely serve as olfactory receptors (Groh et al., 2014; Groh-Lunow et al., 2015). In addition to the behavioral, physiological and genetic aspects, other studies have analyzed the structure of the peripheral and central olfactory pathway in Coenobitidae (Harzsch and Hansson, 2008; Krieger et al., 2010; Brown and Wolff, 2012; Polanska et al., 2012; Wolff et al., 2012; Tuchina et al., 2014).

**Figure 1:**
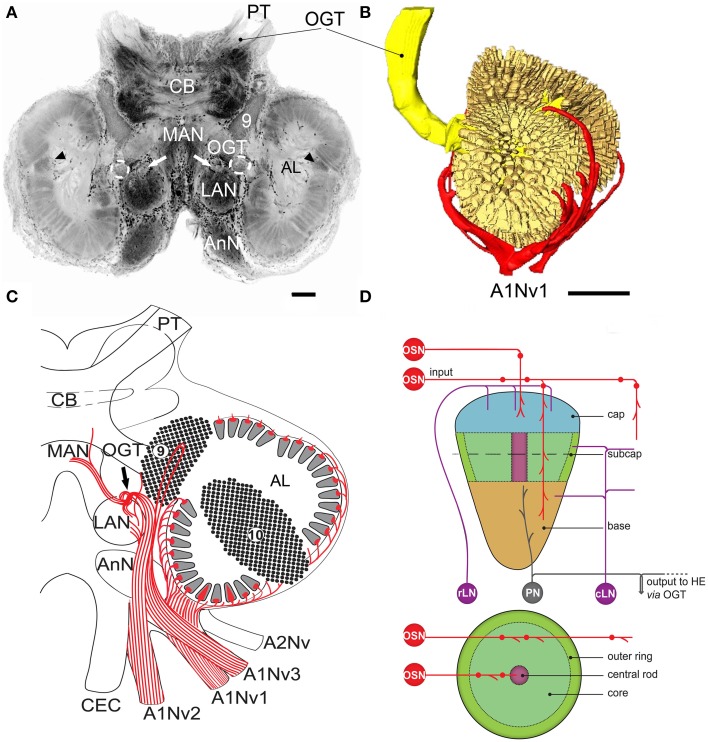
**Overview of *C. clypeatus* median brain as seen in confocal laser scanning microscopy, cLSM (anti-synapsin immunohistochemistry), arrowheads mark non-columnar olfactory neuropils and arrows on **(A,C)** mark a sorting zone for A1Nv2 fibers (see text)**. **(A)** 3D-reconstraction of the antennular lobe, showing olfactory afferents (in red) invading numerous subunits (pale yellow), and axons of the projection neurons (bright yellow) leaving the antennular lobe via olfactory-globular tract and reaching for the higher brain centers; **(B)** Overview of the central projections of antennular nerve (red). **(C)** Schematic representation of the subunits regionalization into cap, subcap, and base in longitudinal section and outer ring, core, and central rod in transverse section. The projection patterns of the olfactory afferents are shown in red; local interneurons in magenta and projection neurons in gray **(D)**. PT, protocerebral tract; OGT, olfactory-globular tract; CB, central body; MAN, LAN, median and lateral antennular neuropil correspondingly; AnN, antenna 2 neuropil; CEC, circumesophageal connectives; A1Nv1, A1Nv2, A1Nv3, branches of the antennular nerve; A2Nv, antenna 2 nerve; AL, antennular lobe; OSN, olfactory sensory neurons; rLN, rim local interneurons; cLN, core local interneurons; PN, projection neurons; HE, hemielipsoid body; 9, cell cluster 9, local interneurons; 10, cell cluster 10, projection neurons. Scale bars: 100 μm on A, 250 μm on B.

In decapod crustaceans, the first pair of antennae, also called “antennules,” plays a major role in the detection of odor molecules (review e.g., Hallberg and Skog, 2011; Derby and Weissburg, 2014). Antennules are equipped with several rows of specialized unimodal olfactory sensilla, the aesthetascs, as well as numerous bimodal mechano- and chemosensory sensilla. At each side of the brain, axons of olfactory sensory neurons (OSNs) associated with aesthetascs on the antennule terminate unilaterally in the corresponding antennular lobe. These primary olfactory centers in the deutocerebrum in most previous studies were termed “olfactory lobes” or “olfactory neuropils” (Sandeman et al., 1992, 1993; Richter et al., 2010; reviews e.g., Schmidt and Mellon, 2011; Harzsch et al., 2012; Derby and Weissburg, 2014; Sandeman et al., 2014). On both sides of the brain, afferent axons emerging from the antennular nerve spread over the surface of the antennular lobe and then terminate in synaptically dense, allantoid-shaped subunits, which in the literature previously were referred to as “olfactory glomeruli” (reviews by Mellon and Alones, 1993; Sandeman et al., 1993; Sandeman and Mellon, 2002; Schachtner et al., 2005; Mellon, 2007; Schmidt and Mellon, 2011; Derby and Weissburg, 2014). The antennular nerve also carries mechanosensory and non-aesthetasc chemosensory neuron axons from bimodal sensilla. These are thought to segregate to two separate regions of the deutocerebrum, the lateral and median antennular neuropils (LAN and MAN; reviews by Harzsch et al., 2012; Strausfeld, 2012; Loesel et al., 2013; Derby and Weissburg, 2014; Sandeman et al., 2014). Within the antennular lobe, its allantoid subunits form an outer layer surrounding a core composed predominantly of connecting fibers of local interneurons and outgoing axons of neurons relaying to higher centers of the brain. Axons from olfactory sensory neurons establish synaptic contacts in these subunits with two classes of olfactory interneurons (see Figure 1): local interneurons (LNs, cell clusters 9 and 11) and projection neurons (PNs, cell cluster 10; see Sandeman et al., 1992), the latter relaying encoded olfactory information to multisensory integration centers in the protocerebrum, the hemiellipsoid bodies (HB) and the terminal medulla (TM; Schmidt and Mellon, 2011; Brown and Wolff, 2012; Wolff et al., 2012).

Previous comparisons of olfactory sensilla and centrally directed olfactory pathways in the terrestrial Coenobitidae and in marine decapods have indicated specific adaptations to the terrestrial lifestyle (Mellon and Reidenbach, 2011). Aesthetascs in adult specimens of *Coenobita* are short and blunt compared to marine hermit crab species such as, *Pagurus bernhardus* (Hansson et al., 2011; Koczan, 2012; Krieger et al., 2012; Tuchina et al., 2014), whereas in planktonic larvae of *Coenobita*, aesthetascs are described as long and slender, typical of marine taxa (Brodie and Harvey, 2001). The paired antennular lobes of Coenobitidae dominate their deutocerebrum, comprising a relatively larger proportion of the entire brain than in marine hermit crabs (Krieger et al., 2010, 2012; Polanska et al., 2012; Harzsch and Hansson, 2008). Whether or not higher centers to which neurons project from the antennular lobes reflect terrestrial adaptations, is not yet resolved although likely. Hemiellipsoid bodies of Coenobitidae are more geometrically precise than in any other crustacean taxon, and their neural architecture is clearly homologous to that of an insect mushroom body (Brown and Wolff, 2012; Wolff et al., 2012; Wolff and Strausfeld, 2015) and their ground pattern organization conforms to that across Arthropoda (Wolff and Strausfeld, 2015).

What might such elaborated hemiellipsoid bodies imply, and how might that elaboration relate to organization of the antennular lobes supplying their afferents? In insects, the most notable elaboration of mushroom bodies is an evolved response to the addition of multimodal inputs, as has occurred in many parasitoid Hymenoptera (Farris and Schulmeister, 2011) or in generalist feeders compared with specialists (Farris and Roberts, 2005). However, mushroom bodies have also obtained enormous elaboration where the peripheral olfactory system alone has become greatly enriched. One example is in Blattoidea, which relies on olfactory cues to exploit spatially distributed resources, implying that olfactory place memory is a pivotal requirement for successful foraging (Mizunami et al., 1993).

The aim of the present study is to further resolve neuronal organization within coenobitid antennular lobes to explore discrete adaptations of the olfactory system that might have been acquired by demands that differ from those of an aquatic habitat, such as territorial behavior and foraging for distant resources. To what degree does the organization of chemosensory and mechanosensory neuron terminals in *C. clypeatus* differ from homologous system in marine decapods? And what might this imply with respect to the next level of olfactory system organization? Insights are provided by the organization of the antennular lobes, particularly the manner in which the peripheral receptor array is represented in the target neuropil. Here we describe these arrangements based on the results of retrograde tracing of afferents from the antennular nerve in combination with Golgi impregnations. Since we performed a mass filling of the antennular nerve and not a single sensillum backfilling, we consider the afferent fibers innervating the antennular lobe to carry olfactory information from the aesthetascs, while the fibers innervating LAN and MAN to carry mechanosensory and possibly chemosensory (non-olfactory) information from bimodal sensilla.

## Materials and methods

### Animals

Adult specimens of *C. clypeatus* (Herbst, 1791; Anomura, Coenobitidae) were obtained from the “Zoologischer Großhandel Peter Hoch” (August Jeanmaire Str. 12, 79183 Waldkirch, Germany; http://www.hoch-rep.com/). The animals were kept in terrariums under 12/12 light cycle, provided with salt and fresh water and food. Animals were anesthetized on ice before the experiments. Experiments were carried out on 20 animals in accordance with the national ethical guidelines (“genehmigungsfreien Versuchsvorhabens nach § 8a Abs.1 und 2 des Tierschutzgesetzes Deutschland vom 18. Mai 2006 BGBl. I S. 1206”), including notification and consent of the responsible administrative authorities of the Max Planck institute for Chemical Ecology in Jena.

### Nomenclature

Although the terms “olfactory lobe,” “olfactory neuropil” and “glomeruli” are ubiquitous to the crustacean neuroanatomical literature (Sandeman et al., 1992 and 1993; reviews e.g., Mellon and Alones, 1993; Sandeman and Mellon, 2002; Schachtner et al., 2005; Mellon, 2007; Schmidt and Mellon, 2011; Derby and Weissburg, 2014; Sandeman et al., 2014), here we suggest using the terms “antennular lobe” and “subunits” instead (see Strausfeld and Hildebrand, 1999; Strausfeld, 2012). Olfactory sensory neurons with their somata are located within the deutocerebral appendages, insect antennae, and crustacean antennules. We suggest that using similar terms reflecting the name of the nerve innervating the neuropil, is appropriate. Further, because the ground pattern organization of the crustacean antennular lobe is considered profoundly different from that of an insect antennal lobe by some of our coauthors, there are anatomical grounds for using the term antennular lobe rather than the less precise appellation olfactory lobe. Another obvious distinction is that unlike afferents to the insect antennular lobe, afferents to the crustacean antennular lobe supply not one but several subunits, a difference ascribed to crustacean olfactory sensory neurons expressing not ligand-gated receptors but ionotropic receptors (see Groh et al., 2014; Groh-Lunow et al., 2015). The neural arrangements within and amongst antennular lobe subunits is consequently distinct from the ground pattern organization of connections within and amongst glomeruli that characterize the insect antennal lobe.

### 3D-reconstruction

In order to make three-dimensional (3D) reconstruction of the antennular lobes, the brain together with the attached antennular nerves was dissected, fixed in 4% paraformaldehyde (PFA) in 0.1 phosphate buffered saline (PBS, pH 7.3) overnight, washed in several changes of PBS, and subsequently post-fixed in 4% glutaraldehyde in PBS overnight at 4°C. Then, the tissues were washed in several changes of PBS, dehydrated in a graded series of ethanol (50, 70, 80, 90, 2 × 100%, for 15 min each) and finally cleared in methyl salicylate and scanned under the confocal laser-scanning microscope Zeiss LSM 510 Meta (cLSM, Carl Zeiss GmbH, Jena, Germany) using z-stacks. Z-series of images were then loaded into AMIRA 5.4.0 (Mercury Systems) and processed using the “Labelfield” editor of Amira. The individual subunits were counted from eight antennular lobes (AL). In five ALs the subunits were sampled via setting “Landmarks” in the 3D image with the help of “Stereo Preferences” and 3D glasses (NVIDIA; 3D VISION Wireless Glasses kit). The cap regions of the subunits were chosen manually for pointing the “Landmarks.” The counting of the pointed “Landmarks” was done automatically by AMIRA. In three ALs the subunits were fully segmented and then counted. Individual subunits were labeled and assigned to “Specific material”; the latter were further counted via “Material Statistics.” The data about volumes of the individual subunits were taken from one antennular lobe, which was fully reconstructed, and further subjected to *T*-test and Shapiro-Wilk normality test.

### Retrograde tracing of the antennular nerve

Anesthetized animals were dissected under the stereo microscope Olympus S2X16. A dorsal part of the carapace was gently removed with scissors, revealing the brain with antennular lobes and the antennular nerves. A crystal of micro-ruby (Dextran, tetramethylrhodamine and biotin, 3000 mW, lysine fixable, Invitrogen) was placed into the stump of the nerve using a glass capillary. Uni- as well as bilateral retrograde tracing experiments were carried out. After 2 h, the brain was dissected and fixed in 4% PFA on 0.1 PBS for 3 h at room temperature or overnight at 4°C, then washed in PBS for at least 2 h and the neurolemma cleaned off with forceps. The fluorescent signal was enhanced by overnight incubation with Streptavidin Alexa Fluor 555 (Invitrogen, 1:1000) in PBS containing 0.1% TritonX (Sigma-Aldrich). In some experiments, the samples were additionally incubated with the monoclonal mouse anti-synapsin “SYNORF1” antiserum (1:20, for 2 days, the antisera were kindly provided by E. Buchner, Universität Würzburg, Germany; see Harzsch and Hansson, 2008), followed by overnight incubation in goat anti-mouse Alexa Fluor 488 secondary antiserum (1:1000, Invitrogen) in order to visualize neuropils, or with Sytox Blue/Orange (1:500, 2 h, Invitrogen) for visualizing cell nuclei. After the incubation, samples were washed in PBS for 2 h, dehydrated in a graded series of ethanol (50, 70, 80, 90, 2 × 100%, for 15 min each), cleared in methyl salicylate and observed under the confocal laser-scanning microscope, Zeiss LSM 510 Meta (cLSM, Carl Zeiss GmbH, Jena, Germany). All images were processed in the cLSM Image Browser and Adobe Photoshop CS5 using global picture enhancement features (brightness/contrast). Single-channel images were generally black-white inverted. All schematic drawings were done in Adobe Illustrator CS5 (Adobe Systems Inc., San Jose, USA). All measurements were done on the fixed tissue.

### Golgi impregnations

Golgi impregnations were performed as described in Wolff et al., 2012. Animals were cooled to 4°C. Antennae, antennules and mouthparts were removed and the rostrum opened while submerged in cold (4°C) fixative comprising 25% glutaraldehyde and 2.5% potassium dichromate containing 10 g sucrose/100 ml admixture. The anterior part of the head containing the brain was next removed from the body and placed in cold fresh fixative for 4 days. Brains were then washed in several changes of 2.5% potassium dichromate (without sucrose) followed by a second chromation (3 days at 4°C) in 99 volumes 2.5% potassium dichromate and 1 volume 1% osmium tetroxide. Tissue was then swirled very briefly (a few seconds) in distilled water and then immersed in 0.75% silver nitrate. Brains were so processed three times to increase the number of fibers impregnated. Thus, after the first silver nitrate treatment, tissue was washed in distilled water and placed again in dichromate-osmium for 2 days and then placed in 0.75% silver nitrate for a further 2 days followed by a third round of impregnation where each impregnation step lasted 24 h before dehydration, embedding in epoxy resin, and serial sectioning.

## Results

### Overview of central projections of the antennular nerve

**Figure 2:**
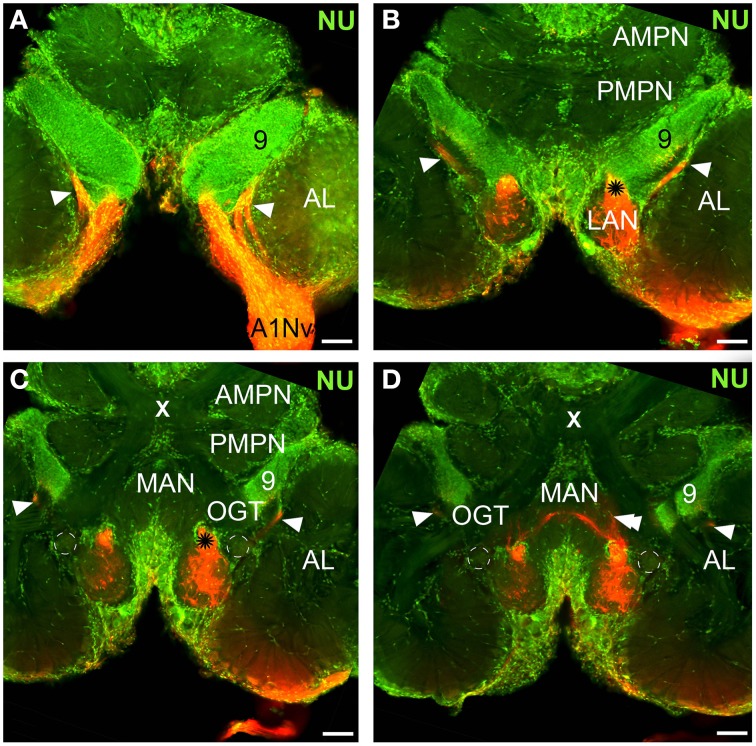
**Overview of the central projections of the antennular nerve, series of cLSM sections with 70 μm step (tracer dextran tetramethylrhodamine-biotin, red; nuclear marker sytox green, NU)**. Asterisks on **(B)**, **(C)** mark a sorting zone for A1Nv2 fibers (see text), arrowheads on **(A–D)** point to the loop formed by A1Nv3 fibers, double arrowheads on D point to the ascending A1Nv3 fibers; dashed lines mark the accessory lobes. AMPN, PMPN, anterior and posterior medial protocerebral neuropils correspondingly; OGT, olfactory-globular tract; MAN, LAN, median and lateral antennular neuropil correspondingly; AL, antennular lobe; 9, cell cluster 9, local interneurons; A1Nv, antennular; X, chiasm of the olfactory globular tract. Scale bars 100 μm.

The antennular nerve in *C. clypeatus* comprises at least three branches (A1Nv1, 2 and 3, see Figure 1C). Despite varying degrees of separation, the nerve branches A1Nv1, 2 and 3 innervate three different neuropils in the deutocerebrum, as also shown by retrograde tracing: the antennular lobe (AL) and the lateral and the median antennular neuropils (LAN and MAN) (Figures 1A,C, 2). These divisions possibly correspond to the separation of the two major sensory inputs from the antennules: unimodal olfactory input to the AL from the aesthetascs; and bimodal mechano-/chemosensory from the non–aesthetasc sensilla to the LAN and MAN (see Schmidt and Mellon, 2011).

### Antennular lobe (AL)

The antennular lobes, which dominate the deutocerebrum, contain the first synaptic relays of the olfactory pathway. Each lobe is composed of dense central neuropil surrounded by a radiating arrangement of about a thousand elongated allantoid-shaped subunits, showing synapsin immunoreactivity (Figure 1A). 3D reconstructions suggest there are well over a thousand subunits per lobe (1065 ± 776; *n* = 8, Figure 1B, Supplementary File), having a range of volumes (40,167 ± 23,887 μm^3^). The number of subunits in a *C. clypeatus* antennular lobe is higher than reported before (799 subunits per AL, Beltz et al., 2003, see Table 1). Nevertheless, the number is still lower than in the close relative of *Coenobita*, the giant robber crab *Birgus latro* (1338, Krieger et al., 2010) or the spiny lobster *P. argus* (1332, Beltz et al., 2003).

**Table 1:** **Number of aesthetascs, OSNs, subunits per AL and aesthetascs/subunits ratio**.

**Species**	**Aesthetasc count**	**OSN count**	**Subunits count**	**Aesthetasc/Subunits ratio**	**Subunit volumes, μm^3^**
***Coenobita clypeatus***	**(519^a^) 358^b^**	**20**,**000^b^**	**(799^a^**) 1065 ± 776^b^	**(0.65^a^**) 0.33^b^	**(154,000^a^)** **40,167 ± 23,887^b^**
*Birgus latro*	(780^d^) 1700^c^	–	1338^c^	(0.58^4^) 1.27^c^	–
*Pagurus bernhardus*	673^b^	33,000^b^	536^b^	1.26^b^	170,000 ± 40,000^e^
*Panulirus argus*	1255^a^	–	1332^a^	0.94^a^	–

a (Beltz et al., 2003);

b (Koczan, 2012) and current manuscript;

c (Krieger et al., 2010);

d (Harms, 1932);

e (Krieger et al., 2012).

Bold indicates data for the species which a subject of the current paper.

**Figure 3:**
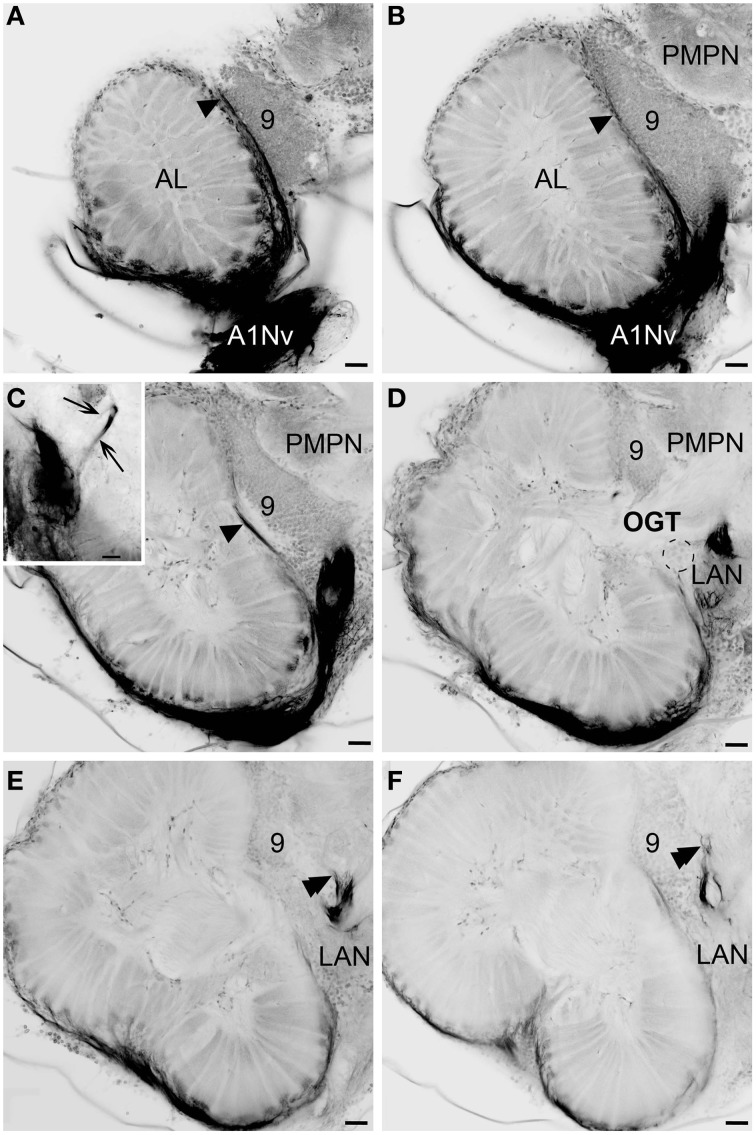
**Innervation of the antennular lobe via the antennal nerve branch A1Nv1, series of cLSM sections with 75 μm step**. Arrowheads on **(A–C)** [arrows on inset in **(C)**] mark the loop formed by A1Nv3 fibers (see text), double arrowheads on **(E,F)** point ascending fibers from LAN, dashed lines on **(D)** mark the accessory lobes. LAN, lateral antennular neuropil; PMPN, posterior medial protocerebral neuropil; AL, antennular lobe; 9, cell cluster 9, local interneurons; OGT, olfactory-globular tract. Scale bars 50 μm.

**Figure 4:**
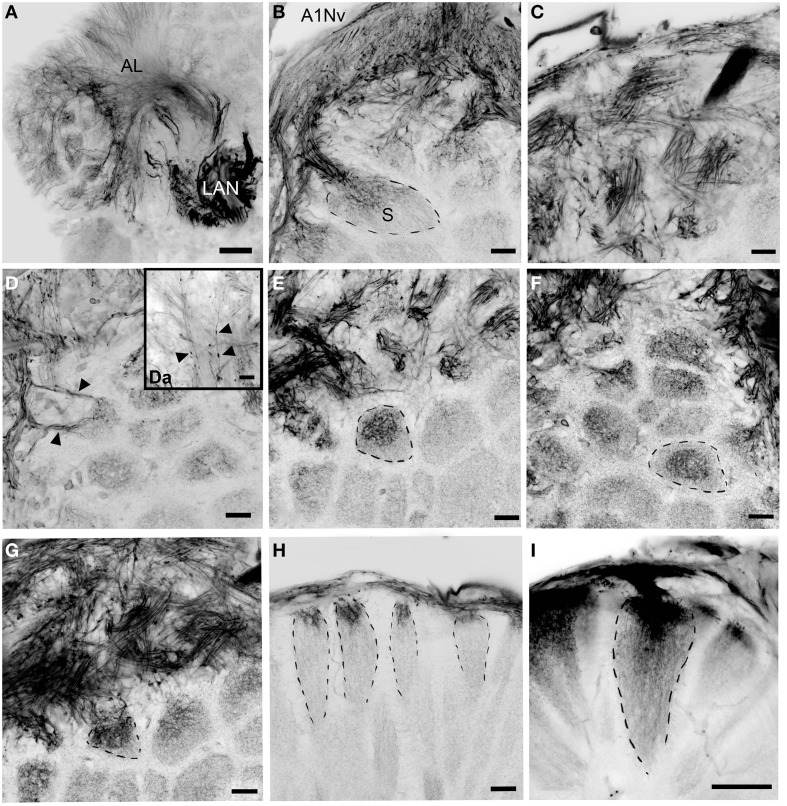
**Details of the antennular lobe innervation, cLSM**. **(A)** Fine afferent fibers invading the antennular lobe and thick fibers innervating LAN. **(B)** Details of the antennular nerve innervating the antennular lobe. **(C)** Bundles of afferents which cross one another in a characteristic pattern before invading the subunits. **(D)** Two bundles of afferent fibers innervation the same subunit (marked with arrowheads). **(Da)** Fine afferent fibers under the higher magnification with numerous swellings (marked with arrowheads). **(E–G)** afferent fibers invading primary the cup of the subunits, frontal view (dashed lines mark the boundaries of single subunits). **(H,I)** Innervation of the subunits, sagittal view. AL, antennular lobe; LAN, lateral antennular neuropil; A1NvM, antennular nerve; S, subunit. Scale bars: 50 μm on A and I, 20 μm on **(B–H)**.

**Figure 5:**
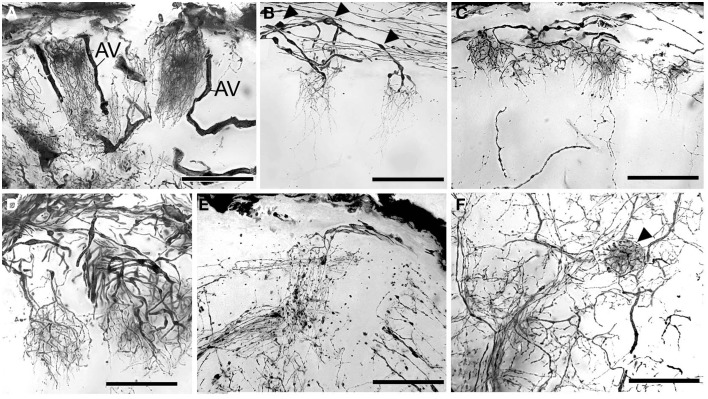
**Golgi impregnations showing the olfactory afferents invading the subunits**. **(A–C)** Two different patterns of subunit innervation (see text), arrowheads on **(B)** mark numerous swellings. (**D)** Olfactory receptor endings, width of cluster on the left corresponds to the cap of the subunit. **(E)** Fine structure of olfactory afferents invading the subunits. **(F)** A single round-shaped subunit found in the antennular lobe (marked with arrowhead). AV, arterial vessel. Scale bars: 100 μm.

The antennular lobe is divided into three sublobes, or domains (Figure 1B), each of which seems equally supplied by afferent fibers from the A1Nv1 branch of the antennular nerve (diameter 100 μm, Figure 3 and Supplementary Figure 1). Most axons in the A1Nv1 are extremely narrow (diameters 0.1–0.5 μm) although a few thicker fibers (diameters 2 μm) contribute to the ipsilateral LAN (Figure 4A). Putative olfactory afferents spread over the surface of the AL where they are arranged in bundles, which cross one another in a characteristic pattern before invading the subunits (Figure 4C). A single subunit is supplied by one or several bundles of axons decorated by numerous button-like swellings (usually several swellings per fiber, with diameters 1–2 μm, Figures 4B–Da, 5). Axons increase in size immediately before they undergo an approximately right angled bend to invade the subunit, providing numerous terminal branches as they do so (Figure 5B). We observed two patterns of innervation: penetration by the terminals down through the full depth of a subunit (Figures 5A,B) or exclusively ending in a superficial layer called the cap/subcap (Figures 4E–I, 5B–D). Occasional spherical neuropils occur deeper within the antennular lobe (Figure 5F). However, neither these spherical subunits, nor the subunits in the accessory lobes (AcL, dashed lines on Figures 1A, 2C,D, 4D) or non-columnar olfactory neuropils (ncON, arrowheads on Figure 1A) receive primary input from olfactory sensory neurons.

### Lateral and median antennular neuropils (LAN and MAN)

**Figure 6:**
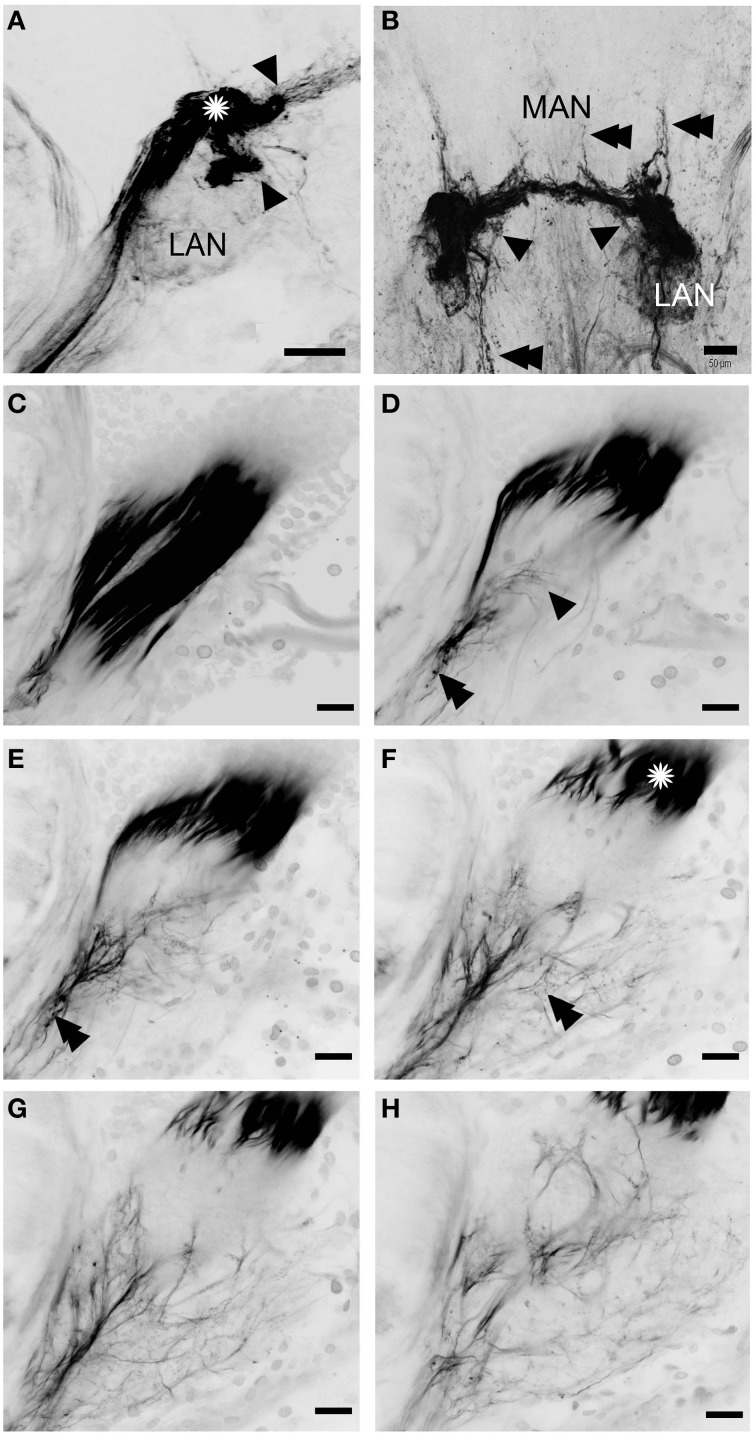
**Innervation of the lateral and median antennular neuropils via the antennal nerve branches A1Nv2 and A1Nv3**. A series of 52 **(A)** and 35 **(B)** cLSM sections merged into one plane with 7.6 and 3.8 μm steps correspondingly. **(C–H)** series of cLSM sections with a step ca. 22 μm. Asterisks on **(A)** and **(F)** mark a sorting zone for A1Nv2 fibers (see text), arrowheads on **(A)** mark the separation of the bundle of afferents into two bunches, on **(B)** point to fibers descending from MAN back to LAN, on **(D)** numerous fine afferents which supply the LAN directly; double arrowheads on **(B)** point to the fibers ascending toward the protocerebrum and descending to the tritocerebrum, on **(D,E)** double arrowheads point to varicosities. MAN, LAN, median and lateral antennular neuropil correspondingly. Scale bars: 50 μm on **(A,B)**, 20 μm on **(C–H)**.

Our observations suggest that axons of putative mechanosensory and non-aesthetasc chemosensory neurons innervate the paired lateral and unpaired median neuropils (LAN and MAN, Figures 1A,C, 2, 6) through the antennular nerve branches A1Nv2 and A1Nv3 (diameter of ca. 75 and 50 μm, respectively). The LAN is an oval-shaped neuropil when viewed horizontally, extending anteriorly toward the arc-shaped MAN (Figures 2C,D). Both LAN and MAN lack allantoid-shaped or glomerular organization, which is a characteristic of olfactory neuropils in general (Hildebrand and Shepherd, 1997; Strausfeld and Hildebrand, 1999), show intense synapsin immunoreactivity (Figure 1A) and appear to be diffusely innervated by the antennular afferents.

The A1Nv2 carries morphologically divergent populations of fibers (with diameters app. 0.1–5 μm) and supplies the LAN and MAN directly, while A1Nv3 fibers (diameters 1–2 μm) extend toward the ipsilateral cluster of olfactory local interneurons (cluster 9) where they form a loop to finally innervate the LAN (arrowheads on Figure 2; inset in Figure 3C). As we observed no varicosities or swellings, there is no evidence of putative synaptic connectivity between A1Nv3 fibers and olfactory interneuron somata. Innervation of LAN is strictly ipsilateral, while fibers to the MAN extend across the midline of the deutocerebrum to the contralateral side (Figures 1C, 6B). The MAN is innervated primarily by a bundle of thick fibers (diameters 2–5 μm) through the A1Nv2, while the LAN receives mostly very thin fibers (0.1–2 μm), emerging directly from the antennular nerve as well as descending from the MAN and A1Nv3.

A sheath of thick fibers from the A1Nv2 primarily invades the region between LAN and MAN forming a node-like bundle, which is devoid of synapsin staining (asterisks in Figures 1A,C, 2B,C, 6A,F), and is further separated into two bunches (arrowheads on Figure 6A), innervating LAN and MAN. A few fibers descend from the MAN back to the LAN (arrowheads on Figure 6B), although most fibers continue toward the midline of the MAN to merge with the afferents from the contralateral side of the brain. Several fibers ascend toward the protocerebrum as well as descend to the tritocerebrum (double arrowheads on Figure 6B). However, it was not possible to identify their destinations. Except for a bunch of thick fibers extending from the node-like bundle, the LAN receives numerous slender afferents directly from the A1Nv2 (arrowhead on Figure 6D). These thin fibers (diameters 0.1–1 μm) branch and form numerous varicosities (double arrowheads on Figures 6D–F). The innervation pattern of both LAN and MAN is complex, which makes it difficult to resolve individual fibers (Supplementary Figure 2).

## Discussion

### Dual chemosensory pathways in the crustacean brain

The first pair of antennae (the antennules) in decapod crustaceans serve mixed, mechano- and chemosensory functions as evidenced by the presence of various physiological types of sensilla (Hallberg et al., 1992; Hallberg and Hansson, 1999; Hallberg and Skog, 2011). Detailed studies include those on several decapods, such as spiny lobsters (genus *Panulirus*; see Gruenert and Ache, 1988; Cate and Derby, 2001), clawed lobsters (genus *Homarus*; see Derby, 1982), crayfish (genus *Procambarus*; Kouyama and Shimozawa, 1982), blue crabs (genus *Callinectes*; Gleeson et al., 1996), and the hermit crabs (genera *Coenobita* and *Pagurus*; Ghiradella et al., 1968a,b). Given this diversity of sensory inputs, the antennular nerve in decapods has been shown to be composed of several discrete bundles, such as medial, dorsal, and lateral (MD, DD, LD) in the spiny lobster *Panulirus argus* representing putative mechano-, non-aesthetasc chemosensory (gustatory), and olfactory afferents (Schmidt et al., 1992; Schmidt and Ache, 1992). We observed a similar organization in the antennular nerve in *C. clypeatus*, although the degree of separation of bundles varies amongst individuals, with all three bundles entering the deutocerebrum separately as A1Nv1, 2, and 3. A1Nv1 supplies mainly the antennular lobe, AL (with a few fibers innervating the LAN, as discussed below), hence carrying putative olfactory afferents and thus likely corresponding to the lateral division (LD) in the spiny lobster antennal nerve. A1Nv2 and A1Nv3 invade the lateral and median antennular neuropils (LAN and MAN), which are known to be processing centers for mechano- and chemosensory (non-aesthetasc) information from the antennules, as well as antennular motor centers (Sandeman et al., 1992; Schmidt and Mellon, 2011). The nerve branches A1Nv2 and 3 likely correspond to the medial and dorsal divisions (MD and DD) of the antennular nerve of the spiny lobster.

Chemosensory input to the decapod brain from the antennules is therefore likely to be represented by dual pathways: aesthetascs to the antennular lobes and bimodal sensilla to the lateral and median antennular neuropils. Schmidt and Mellon (2011) have already pointed out that this anatomical separation corresponds to chemical information being processed as two fundamentally different modes. The first is olfaction *sensu stricto* and is defined as chemoreception of diffusible substances. The second mode has been termed “distributed chemoreception,” and is defined as chemoreception meaning usually but not exclusively contact chemoreception mediated by bimodal sensilla on the antennules but also on other appendages such as biramous second antennae (Schmidt and Mellon, 2011). Schmidt and Mellon (2011) state “the essence of ‘olfaction’ is to provide a detailed representation of the complex chemical environment integrating chemical signals from a variety of sources without reference to the location of stimuli.” In contrast, the essence of the chemoreception mediated by bimodal sensilla, which are located on various appendages, is to form representations of only few key chemicals (such as food-related chemicals and pheromones) within a somatotopic context provided by mechanoreception. The integration of chemo- and mechanosensory information permits pinpointing the location of chemical stimuli.”

### The afferent projections of olfactory sensory neurons

As in other decapod crustaceans (Hallberg et al., 1992; Hallberg and Skog, 2011), in *C. clypeatus* somata of the olfactory sensory neurons belonging to the aesthetascs are arranged as numerous spindle-shaped clusters below the aesthetasc pad (Ghiradella et al., 1968a; Tuchina et al., 2014). We estimate the number of OSNs to be ca. 20,000 per antennula in *C. clypeatus* (Koczan, 2012). This enormous population of sensory neuron axons makes it difficult to resolve the passage of individual sensory neuron fibers from a sensillum to the antennular lobe. The innervation of each lobe via A1Nv1 is strictly ipsilateral which means that comparisons of olfactory information relayed by the right and left antennules is most likely accomplished at the level of the terminal medullas and/or hemiellipsoid bodies (see Harzsch and Hansson, 2008; Wolff et al., 2012). The division of the antennular lobe into two to three large domains (see Supplementary File) is not unique to *C. clypeatus* (Schachtner et al., 2005), but was reported for robber crab *B. latro* (Krieger et al., 2010), marine hermit crab *P. bernhardus* (Krieger et al., 2012), the crayfish *P. clarki*a (Blaustein et al., 1988), the American lobster *Homarus americanus* (Langworthy et al., 1997), and the spiny lobster *P. argus* (Schmidt and Ache, 1997). Based on the number of OSNs in the antennula and the number of subunits in the AL (see Table 1, Results), we conclude that a single subunit in *C. clypeatus* is innervated by approximately 18 sensory neurons, assuming that all subunits are innervated equally. However, each subunit receives inputs from overlapping populations of afferents. The subunits show a remarkable variation in volume, yet nothing is known about whether such differences are a consequence differences in connections between subunits or simply differences on numerical aspects such as profusion of terminal branches and, or, their dimensions. The olfactory subunits in marine hermit crab *P. bernhardus* also show considerable variation in volumes (Krieger et al., 2012; see Table 1). From our tracing studies we observed that a single subunit is innervated from several afferent fibers that also extend terminal branches to neighboring subunits. We thus propose that the great majority of olfactory afferents in *C. clypeatus* supply several subunits. In the spiny lobster *P. argus*, a species intensively studied with regard to its antennular lobe afferents, multiple subunit supply has been suggested to derive from only 10% of all olfactory afferents (Schmidt and Ache, 1992), whereas in the crayfish *P. clarkia*, the few afferent fibers that were filled have been described to terminate only in one subunit (Mellon and Alones, 1993).

The two patterns of subunit innervation described here, afferents terminating exclusively in the cap/subcap region or throughout the entire volume of the subunit down to its base, were also described for *P. argus* (Schmidt and Ache, 1992), whereas studies on *P. clarkia* suggested that labeled olfactory afferents branched throughout the full length of subunit (Mellon and Alones, 1993). The subunits in decapods are known to be regionalized through their depth into a cap, subcap, and base, and in cross-section into a central rod, core, and outer ring (reviews Mellon and Alones, 1993; Sandeman and Mellon, 2002; Schachtner et al., 2005; Mellon, 2007; Schmidt and Mellon, 2011). These zones are defined by the branching patterns of three populations of neural elements: the axon terminals of olfactory sensory neurons, processes of local olfactory interneurons, and the dendritic extensions of output neurons, also known as projection neurons. The presence of two types of receptor terminal morphologies, one extending only as deep as the cap and subcap, the other extending all the way to the subunit base, suggests that olfactory information is segregated, although what this means functionally is debated (see discussion in Schmidt and Mellon, 2011). Numerous varicosities found on the olfactory sensory axons just before each enters its antennular lobe subunit may indicate synapses but could also suggest mitochondrial aggregates in part of the neuron that has a high-energy requirement. Presynaptic inhibition of olfactory afferents was reported earlier for the spiny lobster *P. argus* (Wachowiak and Cohen, 1998), and appears to reduce the amplitude of the action potential delivered into the axon terminals. However, this inhibition occurs within the lobe where it is mediated by local interneurons releasing two inhibitory neurotransmitters, gamma-aminobutyric acid (GABA), and histamine (Wachowiak and Cohen, 1999; Wachowiak et al., 2002). Presynaptic inhibition of afferents is thought to be critical for regulating the sensory input to the antennular lobe. Possibly, some varicosities decorating olfactory sensory axons immediately above the lobe's surface might be indicative of synaptic contacts with rim-local interneurons (rim LNs), a morphological type of LNs which send their processes around the antennular lobe enveloping it, as opposed to core-local interneurons (core LNs) that invade the antennular lobe via the medial foramen (Schachtner et al., 2005; see Figure 9 in Polanska et al., 2012). Rim-local interneurons might also correspond to type 2 AL interneurons according to Wachowiak et al. (1997). So far, we do not have data regarding GABA distribution in the deutocerebrum of *C. clypeatus* although previous studies have identified neuropeptides, such as A-type allatostatins and RF-amides expressed in rim LNs (see Figure 7 in Polanska et al., 2012). Neuropeptides are known to work as neuromodulators in insects and they are often coexpressed together with neurotransmitters (Gutierrez, 2009; Kreissl et al., 2010).

### The afferent projections of non-aesthetasc sensilla

It has been shown in *P. argus* that non-olfactory sensilla provide additional input to the AL (Schmidt et al., 1992). We were unable to demonstrate this for *C. clypeatus* because the antennular nerve was subjected to only mass backfilling. However, as mentioned above, a few fibers from the A1Nv1 branch invade the LAN, which is considered to receive mechanosensory and possibly chemosensory (non-aesthetasc) input from the antennular nerve in other decapod crustaceans (Sandeman et al., 1992). There is no data showing whether the LAN receives primary olfactory input or not, but it is generally assumed that it does not. Thus, the sensory modality of the fibers innervating the LAN from the A1Nv1 branch is open for discussion. However, the LAN also acts as an antennular motor center (Schmidt et al., 1992), and is responsible for antennular movements such as for example, flicking which enhances odor sampling (Koehl, 2011; Nelson et al., 2013). The LAN would thus be expected to receive olfactory information. Remarkably, in *P. argus* the lateral division (LD) of the antennular nerve, a presumed homolog of the AnNv1 in *C. clypeatus*, also supplies both the AL and LAN (Schmidt and Ache, 1992).

Apart from the sensory input, all antennular movements require motor neurons to control muscles. In *P. argus* the somata of antennular motor neurons were labeled via the antennular nerve and a separate motor nerve in two clusters in deutocerebrum (Schmidt and Ache, 1993). Backfilling of the antennular nerve in *C. clypeatus* did not label any cell somata, and hence it is possible that all antennular motor neurons send their axons to antennular muscles via a separate motor nerve that was not backfilled during our experiments.

Mechanosensory and possibly chemosensory (non-aesthetasc) afferents intensively innervate LAN and MAN via antennular nerve branches A1Nv2 and 3. The MAN in *P. argus* receives thick primary fibers from the medial division (MD) of the antennular nerve, whereas the LAN is innervated by fine to medium size fibers via the lateral and dorsal divisions, LD and DD (Schmidt et al., 1992). This general pattern is strikingly similar to that in *C. clypeatus*. However, there are clear differences in the shape of both neuropils and details of their innervation. The LAN in the spiny lobster is a paired bilobed neuropil with a stratified appearance (Schmidt et al., 1992), resembling the second antennal neuropil, AnN, while in *C. clypeatus* the LAN is a paired but spherical neuropil, lacking any divisions into lobes or strata. The explanation for such differences might be in the general morphology of the antennules and the distribution pattern of its sensilla in these two species. The antennule in *P. argus* consists of three basal segments and two equally long, hair-like flagella, medial and lateral, both bearing numerous segments with mechanosensory sensilla (Schmidt et al., 1992). In *C. clypeatus* however the antennule comprises three basal segments and two flagella, which are of quite different size and shape (Ghiradella et al., 1968a). The lateral flagellum in the hermit crab is located dorsally. It is much larger than the medial (ventral) flagellum, bears only few clearly separated segments with mechanosensory sensilla, and the rest of the segments merge to form a plate-like flagellum with the aesthetascs pad. The medial flagellum in *C. clypeatus* is hair-like, has clear segmentation and it is less than a half length of the lateral flagellum (Tuchina et al., 2014). It is therefore possible that change in size and general morphology of the two flagella in *C. clypeatus* is the result of an evolved adaptation to a largely terrestrial habitat and has led to a significant change in the anatomy of the LAN. This hypothesis is corroborated by data on afferent innervation of second antenna neuropil (AnN) in *C. clypeatus*. The AnN is innervated by A2Nv and shows clear stratification resembling segmentally arranged sensilla on the second antenna (Tuchina et al., unpublished observations).

Afferent fibers from antennular nerve branches in *C. clypeatus* extend in parallel to the LAN before invading its neuropil, which is a characteristic also for *P. argus* (Schmidt et al., 1992). Apart from a direct innervation by the antennular nerve, the LAN in *C. clypeatus* also receives fibers descending from the MAN and from the node-like structure between the two neuropils, a region which lack synapsin immunoreactivity (Figure 1A; also see Harzsch and Hansson, 2008) and thus likely to be a sorting zone for the incoming axons before they invade neuropil. A similar node-like structure was described for the spiny lobster (Schmidt et al., 1992). Fibers of A1Nv3 form a loop close to the cluster of local olfactory interneurons before innervating the LAN. However, as there is no evidence for synapses between the A1Nv3 fibers and local interneurons, the possible function of this pathway is not clear.

The MAN is suggested to receive primary mechanosensory information (mainly from the statocysts) and thus function as an equilibrium center (Schmidt et al., 1992). In the spiny lobster, the MAN is a comparatively large neuropil, unpaired and irregular in form, but symmetrical around the midline of the deutocerebrum (Sandeman et al., 1992; Schmidt et al., 1992). This general morphology is quite similar to that in *C. clypeatus*. Unlike the LAN and the AL, the innervation pattern of the MAN is not strictly ipsilateral because some fibers extend across the brain's midline to reach the contralateral half of the MAN, a pattern that fits the hypothesis of the MAN being an equilibrium center (Schmidt and Ache, 1993). A few fibers descending from the MAN to the tritocerebrum have been also described for the spiny lobster, where they are assumed to target the tegumentary neuropil (Schmidt et al., 1992), which in decapods is innervated by afferents from the dorsal carapace (Sandeman et al., 1992).

## Conclusions

The central projections of antennular nerves in *C. clypeatus* in our paper were compared mainly with well-studied descriptions of the aquatic spiny lobster, *Panulirus argus*. Similarities of *C. clypeatus* and its remote marine relative, *P. argus* include the division of the antennular nerve into three bundles: one possibly carries olfactory information from the aesthetascs to the antennular lobe, the others relaying chemical and mechanosensory information from bimodal sensilla to the lateral and median antennular neuropils. In both species, putative olfactory afferents envelope the AL and invade its subunits with two clear patterns of innervation. However, there are also obvious distinctions that possibly reflect differences of terrestrial and marine habitats, one difference being the shape and innervation pattern of the LAN by putative mechanosensors. We propose that the major distinction with respect to the innervation pattern seen in the spiny lobster is that the majority of putative olfactory sensory axons in *C. clypeatus* invade multiple subunits. However, overall, there seem to be little major differences of the antennular lobes between the terrestrial and aquatic species. Indeed, this may not be surprising considering that *C. clypeatus* begins its life as an aquatic animal. A search for terrestrial adaptations in the olfactory system of *C. clypeatus* would greatly profit from the comparison with closely related Anomuran species (such as for example with aquatic species *Pagurus bernhardus*) but unfortunately there is no data available on the central projections of antennular afferents in *P. bernhardus*.

In order to compare our current knowledge on the similarities and differences between terrestrial *C. clypeatus* and aquatic *P. argus* and *P. bernhardus*, we can highlight the following points:

- Aesthetascs in *C. clypeatus* are short and blunt compared to marine hermit crab species *P. bernhardus* and *P. argus* (Ghiradella et al., 1968a,b; Derby et al., 2001; Koczan, 2012; Krieger et al., 2012; Tuchina et al., 2014); number of aesthetascs varies, being the least in *C. clypeatus* (358 per antennula compared to 673 in *P. bernhardus* and 1255 in *P. argus*) and the number of OSNs in *C. clypeatus* is less than in *P. bernhardus* (20,000 vs. 33,000 per antennula) (Beltz et al., 2003; Koczan, 2012);
- Antennular transcriptomes of *C. clypeatus* and *P. bernhardus* do not show a significant difference in genetic makeup connected to terrestrialization. *C. clypeatus* and *P. bernhardus* have a similar set of ionotropic receptors (IRs) (Groh et al., 2014; Groh-Lunow et al., 2015);
- Antennular nerve in *C. clypeatus* and *P. argus* is divided into three bundles, supplying different neuropils in the deutocerebrum (Schmidt et al., 1992);
- There seem to be no direct correlation between terrestrial habitat and the number of olfactory subunits in the AL: 1065 in *C. clypeatus* vs. 536—in *P. bernhardus* and 1332—in *P. argus*; subunits vary greatly in volumes within the AL (Beltz et al., 2003; Koczan, 2012);
- Putative olfactory sensory axons in *C. clypeatus* and *P. argus* envelope the AL and invade its subunits with two clear patterns of innervation; majority of putative olfactory sensory axons in *C. clypeatus* invade multiple subunits, as well as in *P. argus* (Schmidt and Ache, 1992).
- There are clear differences in the shape and innervation pattern of the LAN in *C. clypeatus* compared to *P. bernhardus* and *P. argus*; No such differences found for MAN (Schmidt et al., 1992);
- The multisensory integration centers, namely the hemiellipsoid bodies, in *C. clypeatus* and *P. bernhardus* show similar subdivision into a cap and two core neuropils, however this division is more pronounced in *C. clypeatus*; the size of the hemiellipsoid neuropils is bigger in *C. clypeatus* than its marine counterpart (Krieger et al., 2012; Wolff et al., 2012).

Thus, future studies are needed to resolve once and for all the still outstanding question as to whether a crustacean living much of its life in air truly differs from one that lives its whole life in water, and whether living on land has resulted in any obvious evolved adaptations to the chemosensory system and the brain it supplies.

### Conflict of interest statement

The authors declare that the research was conducted in the absence of any commercial or financial relationships that could be construed as a potential conflict of interest.
