# Supplementary figures and images for: Central projections of antennular chemosensory and mechanosensory afferents in the brain of the terrestrial hermit crab (Coenobita clypeatus; Coenobitidae, Anomura)
Source: Front Neuroanat. 2015 Jul 15;9:94. doi: 10.3389/fnana.2015.00094 (PMC4502362; doi:10.3389/fnana.2015.00094)

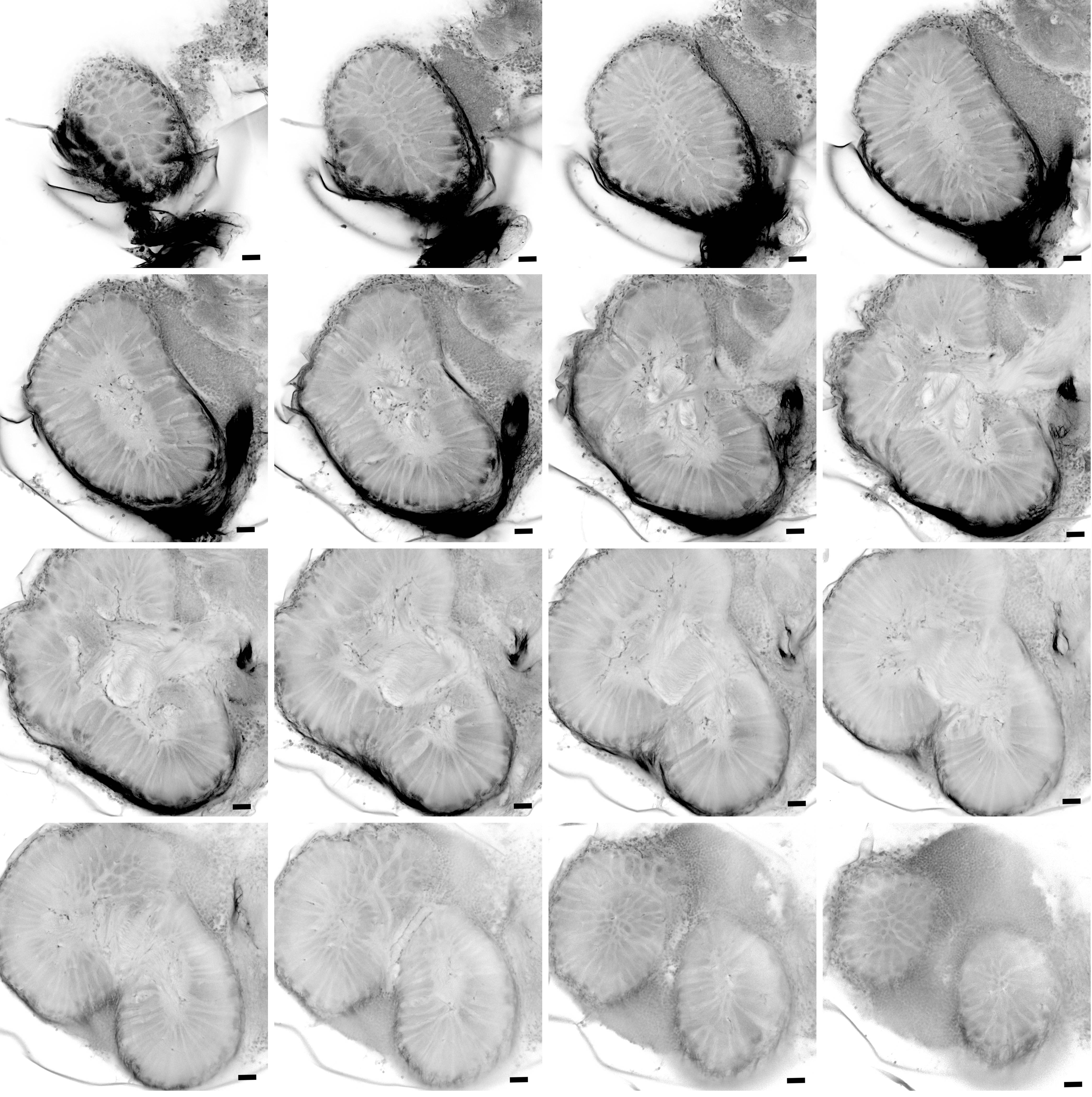

Supplement: Supplementary file 2 [file Image1.JPEG]

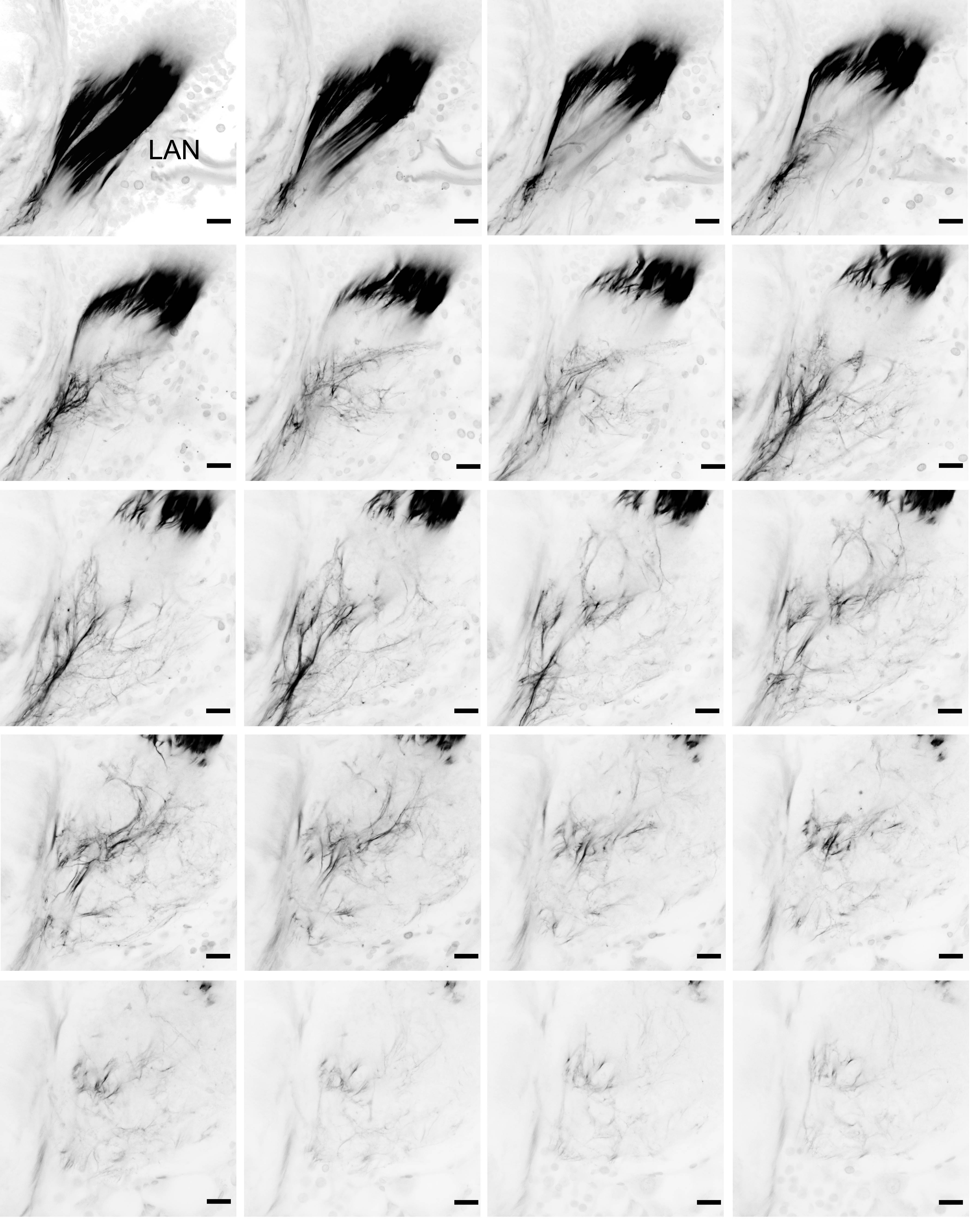

Supplement: Supplementary file 3 [file Image2.JPEG]
